# Text mining facilitates database curation - extraction of mutation-disease associations from Bio-medical literature

**DOI:** 10.1186/s12859-015-0609-x

**Published:** 2015-06-06

**Authors:** Komandur Elayavilli Ravikumar, Kavishwar B. Wagholikar, Dingcheng Li, Jean-Pierre Kocher, Hongfang Liu

**Affiliations:** Department of Health Sciences Research, Mayo Clinic College of Medicine, 200 First St SW, Harvick 3rd, Rochester, MN 55905 USA

**Keywords:** Mutation mining, Text mining, Protein mutation disease association

## Abstract

**Background:**

Advances in the next generation sequencing technology has accelerated the pace of individualized medicine (IM), which aims to incorporate genetic/genomic information into medicine. One immediate need in interpreting sequencing data is the assembly of information about genetic variants and their corresponding associations with other entities (e.g., diseases or medications). Even with dedicated effort to capture such information in biological databases, much of this information remains ‘locked’ in the unstructured text of biomedical publications. There is a substantial lag between the publication and the subsequent abstraction of such information into databases. Multiple text mining systems have been developed, but most of them focus on the sentence level association extraction with performance evaluation based on gold standard text annotations specifically prepared for text mining systems.

**Results:**

We developed and evaluated a text mining system, MutD, which extracts protein mutation-disease associations from MEDLINE abstracts by incorporating discourse level analysis, using a benchmark data set extracted from curated database records. MutD achieves an F-measure of 64.3 % for reconstructing protein mutation disease associations in curated database records. Discourse level analysis component of MutD contributed to a gain of more than 10 % in F-measure when compared against the sentence level association extraction. Our error analysis indicates that 23 of the 64 precision errors are true associations that were not captured by database curators and 68 of the 113 recall errors are caused by the absence of associated disease entities in the abstract. After adjusting for the defects in the curated database, the revised F-measure of MutD in association detection reaches 81.5 %.

**Conclusions:**

Our quantitative analysis reveals that MutD can effectively extract protein mutation disease associations when benchmarking based on curated database records. The analysis also demonstrates that incorporating discourse level analysis significantly improved the performance of extracting the protein-mutation-disease association. Future work includes the extension of MutD for full text articles.

## Background

Recent advances in next generation sequencing technology has accelerated the pace of individualized medicine (IM), which aims to incorporate genetic/genomic information into medicine [1]. One immediate need in interpreting sequencing data is the assembly of information about genetic variants especially mutations in coding regions (i.e., protein mutations) and their associations with diseases. Such information has been catalogued in multiple biomedical databases such as ClinVar [2], OMIM [3], or UniProtKB [4]. Peterson et al., 2013 [5] list nearly 21 resources containing protein mutation disease associations. Even with dedicated effort in capturing such information in biomedical databases, much of this information still remains ‘locked’ in the unstructured text of biomedical publications. Additionally, the interpretation of “novel” variants has been a significant task since some of the variants may not be “novel” which are already published in the literature. For example, the latest evidence indicates that PSEN1, p.E318G variant is associated with the development of early-onset of Alzheimer’s disease (AD) for APOE-ε4 carriers [6–10]. OMIM has not been updated with this clinically relevant finding. Instead OMIM states “the E318G change is a polymorphism with uncertain clinical significance” based on the 2005 publication [11]. Similarly, many of these databases including UniProtKB and NCBI ClinVar fail to report the significance of the PSEN1, p.E318G variant with early onset AD. Thus, the development of text mining approaches to accelerate the process of assembling IM knowledge in published literature is necessary.

Text mining techniques can be potentially applied to mitigate such information lag [12]. Multiple text mining systems have been developed for extracting mutation events (e.g., Mutation-Finder [13] and EMU [14], tmVAR [15]); as well as relation extraction to associate mutation events to other entities such as proteins, (e.g., MEMA [16], MuteXt [17], MuGeX [18] and Mutation-GraB [19]), genes (e.g., Polysearch [20]), diseases (e.g., SNPShot [21]), and drugs (e.g., SNPShot). Most of these systems use regular expressions to detect mutation mentions form bio-medical text but associations between mutations and other entities are based on simple co-occurrence information. For example, SNPShot extracts various binary relations between genes, variants, drugs, diseases and drug reactions using simple co-occurrence statistics and normalize entities to databases such as EntrezGene [22], PharmGKB [23], or PubChem [24].

MEMA [16] and MuteXt [17] applied word distance metric to retain the right protein-mutation pairs extracted based on sentence co-occurrence.; Mutation-GraB [19] applied weighted graph-based traversal to resolve ambiguity and retain only the protein-mutation pairs that have the shortest path between them. On the other hand, Doughty et al. [14] have proposed the use of sequence information to validate the associations. More sophisticated approaches based on grammar parsing have been extensively employed for relation extraction in the NLP shared task. For example, Open Mutation Miner [25] attempts to extract the impact of mutation on protein function and its properties such as kinetics and stability using a rule-based approach based on shallow parsing. Some recent studies on automatic linking of protein mutations and diseases consider advanced linguistic features such as dependency parse graphs [26, 27].

### Rationale

Most of the previous studies discussed above [17, 18,20, 21] have estimated their text mining performance against text-based gold standard annotations and not against human curated gold standard annotations in the databases. While the text based gold standard annotations consider only the information given in the document for annotation, database annotations may infer information from multiple documents relevant to the task for curation besides the curator’s domain knowledge. Hence evaluating text mining systems against biomedical text gold standards does not estimate the “practical utility” of these systems for database curation task. In this section, we briefly discuss the rationale behind this work and the issues that we attempt to address in the current study.

#### Biomedical database curation workflow and the role of text mining

Database curation broadly involves the following steps [28] generally followed by human curators with the required domain knowledge.

Step 1 **Problem and curation guideline definition** - Define the problem domain selected for curation and its guidelines,
Step 2 **Collection of literature pool** – Collect the seed literature pool through appropriate choice of search terms using information retrieval engines such as PubMed.
Step 3 **Retrieval of relevant article** – Retrieve the relevant abstracts/articles from the literature tool using API such as Entrez e-utils in PubMed.
Step 4 **Identification of relevant evidence sentences** – Manually read the literature and identify the evidence sentences in the individual abstract/article based on their relevance to the target problem considered for curation.
Step 5 **Identification of entities** – Identify and mark up the entity mentions in the text.
Step 6 **Normalization of entity mentions to database concepts** - Normalize the entity mentions in the text to ontological concepts or database identifiers.
Step 7 **Identification of relations between entities** - Extract the relations between the entity mentions both within and across sentences.
Step 8 **Store the abstracted concepts and relation in the database** – This is the final step that involves transformation of information in natural language to structured representation in flat file such as XML or in a relational database.

The major bottleneck in the database curation workflow is the manual effort required in each of these steps. Text mining systems have been used to assist the curators to accelerate the curation workflow, mainly for the initial steps from 1 to 4. The performance of the existing tools is unsatisfactory for the latter steps from 5 to 7. Steps 5–7 involve normalization and inferences, which require synthesis of information across sentences, documents, and domain knowledge sources such as bio-medical ontologies.

**Fig. 1:**
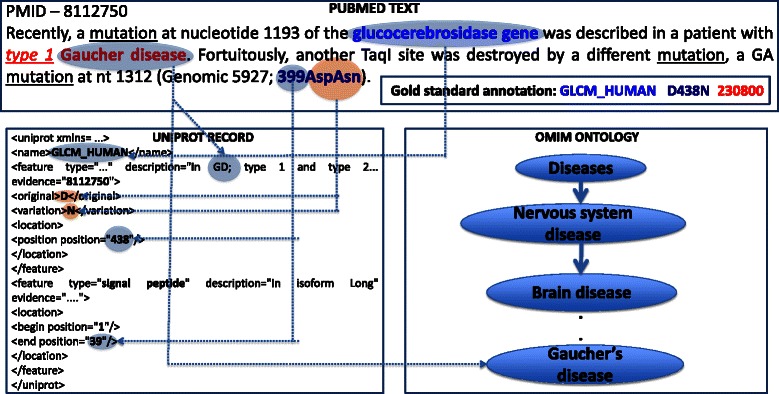
Role of human inference in database curation

Figure 1 illustrates two major inference challenges: linguistic inference and expert inference using two sentences from an abstract (PMID-8112750) describing the association between a point mutation “Asp399Asn” of “glucocerebrosidase gene” and “Gaucher’s disease”. While the first sentence has the mentions of gene/protein and disease, it is the second sentence that provides the site of mutation information. Linguistic inference is required to connect the two pieces of information mentioned in different sentences to infer the role of mutation in a disease. Current text mining tools fall short of making such linguistic inferences. Prior to relation extraction, normalization of the entities to the ontological/database definitions requires inference predominantly based on the background knowledge and the linguistic inference drawn from the description in the current text. The state of the art of entity normalization, particularly the gene and disease has been steadily increasing to acceptable levels thanks to the organization of series of shared tasks such as BioCreative [29, 30]. However, normalization of the mutation site mentions in the text to the sequence in biomedical databases has been a challenge. It requires inference by the curation expert to judge whether the mutation residue position mentioned in the text accounts for the signal peptide region. In the example shown in Figure 1 the position of mutation Asp to Asn is mentioned as “399” in the text. However, the gold standard annotation in UniProtKB mentions the site of mutation to be “438”. The curator has utilized his/her biological knowledge to infer that the sequence reported in the paper does not include signal peptide region (a total of 39 residues). The length of signal peptide region, 39 is added to the position mentioned in the text, 399, which gives the final position, 438, mentioned in the gold standard. Such expert inference capability is beyond any text mining system.

Leveraging the existing knowledgebase annotations for text mining often referred to as weakly supervised/distant supervision learning has been on the rise in other domains [31, 32]. Such distant supervision approaches [33, 34] were explored in a limited context for learning patterns relating protein and its sites mentioned in a single sentence using multiple database annotations such as UniProtKB and PDB.

In this paper, we present a text mining system, MutD, which extracts protein mutation-disease associations from MEDLINE abstracts by incorporating discourse level analysis. We evaluated the performance of MutD to extract mutation disease relations against human curated annotations in UniProtKB as the gold standard. In the following sections, we describe tools and resources, our discourse level analysis approach, and experimental methods.

## Methods

### Resources and Tools

The system developed for this study (MutD) was implemented under Apache Unstructured Information Management Architecture (UIMA) [35]. We used the Unified Medical Language System (UMLS) (version 2012 AB) [36], BioThesaurus (V2.1) [37], Comparative Toxicogenomics Database (CTD)’s [38], and merged disease vocabulary (MEDIC) [39] ontology as terminology sources for recognizing diseases and proteins mentioned in the text. BioTagger-GM [40] is used for dictionary lookup. We used Stanford parser [41] to generate the dependency parse representation from individual sentences. For entity recognition and normalization, in addition to customized BioTagger-GM [40] we also used existing systems, MutationFinder [13], PubTator [42] and GeNo [43]. We used UniProtKB to extract curated ternary relations to form a gold standard data set to develop, refine and evaluate the system. The following provides a brief background of each of resources and systems.

#### UIMA

Apache UIMA [35], is a data-driven architecture where components communicate with each other through Common Analysis System (CAS) containing annotations. Each annotation is an instance of a given type defined by a specified hierarchical type system.

#### UniprotKB

UniProtKB [4] is a protein database partially curated by experts, consisting of two sections: SwissProt where entries have been manually annotated and TrEMBL where entries are automatically annotated. The manual annotation process of an entry in UniProtKB involves detailed analysis of the protein sequence with the scientific literature being the primary evidence. For this study, we specifically use the curated information about mutation and its role in diseases. Only the SwissProt entries are used to automatically generate the gold standard data set.

#### BioThesaurus

BioThesaurus [37] is a web-based system designed to map a comprehensive collection of protein and gene names to protein records in UniProtKB [4]. Currently covering four million proteins, BioThesaurus consists of over 6 million names extracted from multiple molecular biological databases according to the database cross-references in iProClass [44]. The data is downloadable from the iProLINK [45].

#### MEDIC

MEDIC is a comprehensive collection of disease names from two major resources namely MeSH [15, 36] and OMIM [3]. The OMIM entries are mapped to hierarchical entries in MeSH ontology. This results in not only the integration of both these resources but provides a hierarchical structure to OMIM ontology as well. MEDIC consists of 9700 unique diseases with close to 67, 000 terms that includes synonyms.

#### BioTagger-GM

BioTagger-GM [40] is a gene recognition system, which takes a hybrid approach to recognize gene names. In the first step, the system combines dictionary lookup with machine learning to detect gene names. Subsequently heuristics were used to filter false positives. The system finally performs a voting on the output of all methods, and builds a consensus among them. The system achieved F-measure of over 85 % for the gene mention task, and ~78 % for the gene normalization task. Here gene mention refers to the detection of the spans of gene names in the text, while gene normalization refers to associating a specific gene mention to a unique Entrez gene symbol. BioTagger-GM is the state of the art system for gene mention and normalization.

#### MutationFinder (MF)

MutationFinder [13] is an open-source, high-performance information extraction system that extracts mentions of point mutations from free text. MutationFinder applies a set of approximately 700 regular expressions to identify mutation mentions in the input text. On blind test data, the precision of MF is 98.4 % and recall 81.9 %, when extracting point mutation mentions. We used MutationFinder version 1.1 for this study, which is implemented in UIMA.

#### GeNo

GeNo [43] is a gene normalization tool that uses a set of symbolic and statistical methods by fully relying on publicly available software and data resources, including extensive background knowledge based on semantic profiling. GeNO achieved a F-measure of 86.4 % for the gene normalization task on the BioCreative-II [29, 30] test set. GeNO is available as a remotely employable UIMA Analysis Engine (AE). This AE bundles the gene mapper with key components like named entity recognition component JNET [46].

#### PubTator

PubTator [42] is a web-based service from NCBI that delivers entity annotations such as Gene, Mutation, Disease, Species and Chemical for PubMed abstracts. The entities are further normalized to ontological definitions where genes are normalized to Entrez Gene Id, disease and chemical names are normalized to MeSH ID and species to NCBI taxonomy. PubTator is an ensemble of state of the art algorithms for entity detection namely GenTUKit [47] for detecting gene mentions, GenNorm [48] for Gene normalization and SR4GN [49] for species detection and normalization and DNorm [50] for disease entity mentions and normalization.

### System implementation

**Fig. 2:**
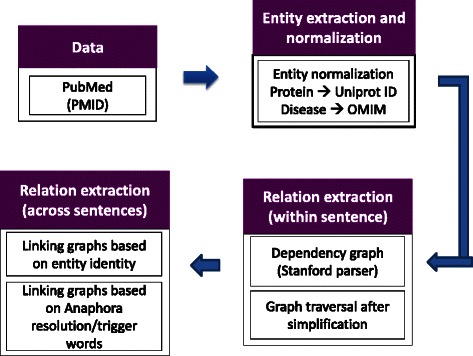
System architecture

Figure 2 illustrates the overall system architecture implemented for this study. The system includes components for retrieval of abstracts, sentence detection, tokenization, lexical normalization, dictionary lookup for entity normalization, and relation extraction. The following sections briefly describe the details of each of the components.

#### Retrieval

The pipeline starts with the retrieval of PubMed abstracts for a given query (PMID in the current work) using the Entrez e-utils web service [51].

#### Preprocessing

The system includes a series of pre-processing modules such as sentence splitting and tokenization using OpenNLP [52]. It also includes a lexical normalization component, which converts words to a canonical form using the Lexical variant generator (LVG) [53] provided by the National Library of Medicine (NLM).

#### Term identification

The term identification component has modules to recognize protein, point mutations, and disease terms from PubMed abstracts. We retrieved the entity annotations: protein/gene, mutation and disease for all the PubMed abstracts considered for this study using the web service of PubTator [42] through the web service provided by the tool. The protein/gene, mutation, and disease from PubTator normalized to Entrez Gene ID, dbSNP/MEDIC, and MeSH respectively. For this study we considered only the point mutations (substitutions) annotated by tmVAR of PubTator. Besides PubTator, we also used entity recognition for protein from the Entrez/UniprotKB, applied within a BioTagger-GM [40] framework. For mutations, we also use MutationFinder [13] to detect protein point mutations from the text. We ran each of these tools individually and finally retain all the normalized annotations from the combination of a selection of state of the art tools such as PubTator, GeNO, BioTagger-GM, and Mutation-Finder. Wherever there is disagreement between the entity boundaries we considered the longest span of the entity.

#### Detection of abbreviations

Abbreviations are quite often used in biomedical literature. Entity detection tools may identify abbreviations and the associated definitions (protein/disease). We use the Schwartz algorithm [54] to detect abbreviations. If the long form is identified as an entity, we also assign the same semantic type and ontology id to the short form within the scope of the abstract.

#### Graph traversal in the dependency parse graph representation

**Fig. 3:**
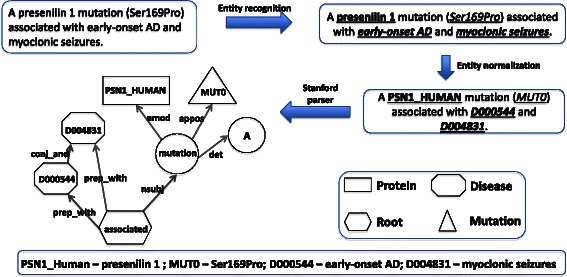
Steps in dependency parse graph traversal

Post entity extraction we simplify the sentences by replacing the actual entities in the sentence with the normalized entities. For example consider the following sentence from an abstract: “A **presenilin 1** mutation (*Ser169Pro*) associated with ***early-onset AD*** and ***myoclonic seizures***”. We replace the actual entity mentions with either Ontology IDs or symbols, which can be retraced in the text. Protein mention such as “presenilin 1” in the above sentence is replaced by its Uniprot ID (**PSN1_HUMAN**). Similarly, disease mentions such as “early onset AD” and “myoclonic seizures” are respectively replaced by MeSH Ids “***D000544***” and “***D004831***”. We replace the mutation mentions with the text “MUT” followed by an incremental index number as they occur in the text. If two mutations are identical, they are replaced by the same index. For example in the example sentence, the mention of Ser169Pro is replaced with *MUT0*. The original sentence after replacements is simplified to: “A **PSN1_HUMAN** mutation (*MUT0*) associated with ***D000544*** and ***D004831***.” We then process the simplified sentence through Stanford dependency parser [41] and obtain the dependency graph, which captures the dependencies between the words in the modified sentence. We use the collapsed dependencies graph representation, which propagates the conjunction dependencies and collapses the dependencies involving prepositions. The approach is similar to the one proposed by Coulet et al. 2010 [26]. During the graph traversal, we assume that if the three target entities (i.e. protein, mutation and disease) share a common node, they are related to each other. Figure 3 illustrate all the individual steps in relationship extraction through graph traversal using an example sentence involving all the three entities.

#### Extra-Sentential processing

Quite often we do not find all the three entities (i.e. protein, mutation, and disease) occur in a single sentence. They are often located in different sentences. To handle such cases we developed methods to associate entities across multiple sentences. Our approach includes two steps:

##### Linking dependency graphs from multiple sentences

**Fig. 4:**
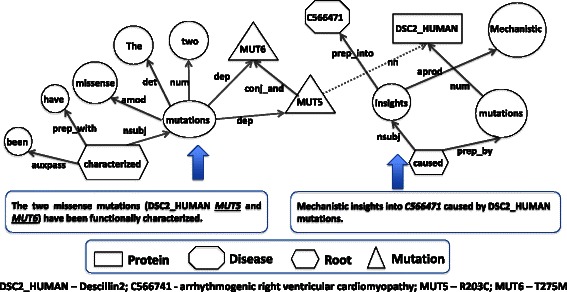
Linking dependency graphs on entity identity

As the first step towards extracting information from different sentences, we link dependency graphs across sentences based on entity identifiers. If dependency graphs from two or more individual sentences share the same entity, we link the graphs. Consider the following two sentences from an abstract (PMID: 21062920) where the entity mentions are replaced with database identifiers/symbols. “Mechanistic insights into ***C566471*** caused by **DSC2_HUMAN** mutations.” and “The two missense mutations (**DSC2_HUMAN** *MUT5* and *MUT6*) have been functionally characterized …” The dependency parse graph traversal for the first sentence (shown in Figure 4) yields only the relationship between the disease *arrhythmogenic right ventricular cardiomyopathy* (MesH: ***C566471***) and the protein desmocollin-2 (UniProt ID: **DSC2_HUMAN**). No point mutation is mentioned within the same sentence. However in one of the subsequent sentence there is a mention of the same protein and its two point mutations. The relation between the two mutations R203C (replaced with MUT5) and *T275M* (replaced with *MUT6*) and the protein (**DSC2_HUMAN**) is extracted by the dependency parse graph traversal as shown in Fig. 4. Since the protein **DSC2_HUMAN** is common between the two graphs we linked these two graphs, which yield a connection between the three entities yielding two ternary relations namely < **DSC2_HUMAN**, *R203C*, ***C566471*** > and < **DSC2_HUMAN**, *T275M*, ***C566471*** > .

##### Linking dependency graphs across sentences using anaphora and trigger words

**Fig. 5:**
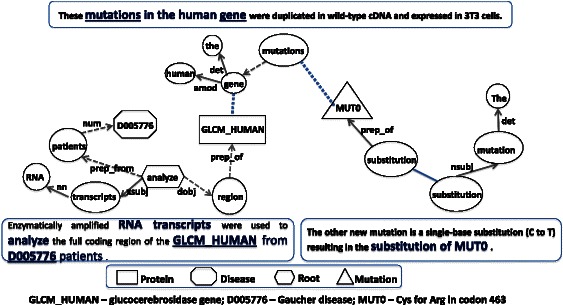
Linking dependency graphs based on discourse analysis

In scientific discourse, it is quite common to use anaphora to refer to entities mentioned in other portions of the text. In this work, we consider linking dependency parse graphs from multiple sentences through anaphora resolution. We adapted the anaphora resolution system that is described in Ravikumar et al., 2013 [55]. Besides anaphora, we observed a lot of other terms, which we call, 'trigger words' that refer to entities that are mentioned in earlier sentences or one of the subsequent sentences. We treat such trigger words (e.g. mutation, mutant, variations, variants, mutational, patient, phenotype) as anaphoric though they may not be considered anaphora category in strict grammar sense. Figure 5 illustrate how anaphoric references are used to link two dependency graphs from different sentences.

### Post-processing

A few post-processing steps are implemented after extracting these relations. We replace the modified mutations with the original entities during this step. Then we separate the three individual components of point mutations from PubTator namely the wild type residue, the mutant residue and the position of the residue in the amino acid sequence. Next, we map the mutant and the wild type residue from its single letter or full name notation to its three-letter notation. We also map the disease MeSH Ids to OMIM Ids through MEDIC ontology [39] available in CTD [38]. Mapping disease terms to OMIM is essential as the disease annotation in UniProtKB is normalized to OMIM entries. For example, the ternary relation < **DSC2_HUMAN**, *R203C*, ***C566471*** > extracted from the text, will be finally transformed to < **DSC2_HUMAN**, *Arg, 203, 275, Cys*, ***610476*** > where **DSC2_HUMAN** is UniProtKB ID, Arg is the wild type residue, 203 represents the residue position, 275 the normalized residue position after accounting for the signal peptide region, Cys is the mutant residue and ***610476*** is the OMIM Id for the disease *arrhythmogenic right ventricular cardiomyopathy.* If there is more than one OMIM Id associated with a MeSH Id, we retain all the OMIM Ids for that output.

### Experiments

**Fig. 6:**
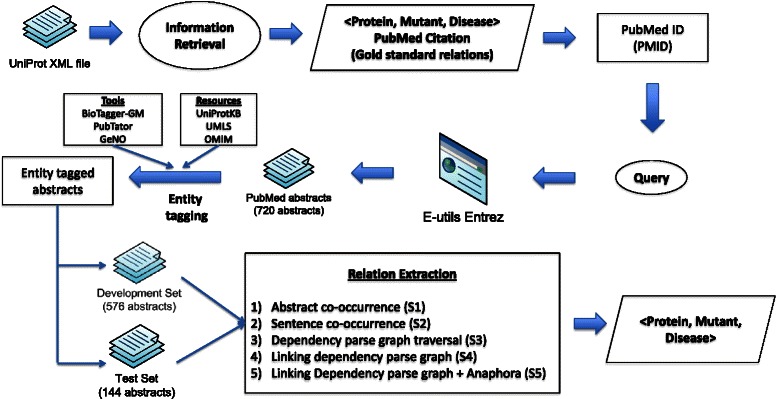
Experimental design

Figure 6 illustrates the experimental workflow that we adopted in the study. We first extracted gold standard instances from curated database records in UniProtKB. Specifically, we used SwissProt, the manually curated portion in UniProtKB, to extract protein mutation disease associations that are used as a gold standard.

**Fig. 7:**
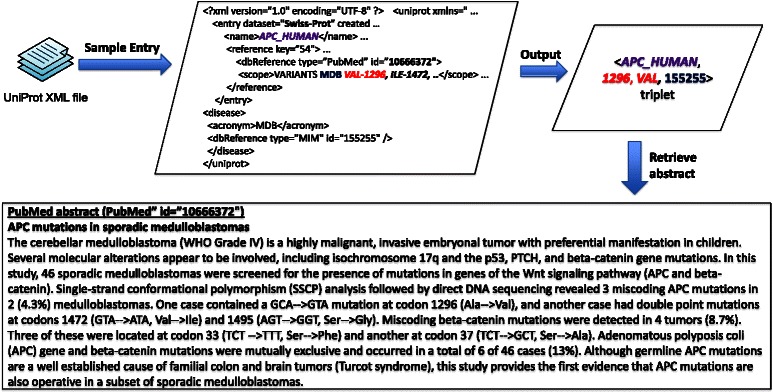
Extraction of gold standard from UniProtKB

Figure 7 shows an example illustrating the steps involved in extracting gold standard instances. Specifically, we extract UniProtKB ID (**APC_HUMAN**), wild type (Ala), its position in the sequence (1296), mutant residue (Val), OMIM ID (***155255***, i.e., ***Medulloblastoma***) from the curated portion of UniProtKB (i.e., SwissProt) and the cited PubMed Id (PMID - 10666372). If the sequence annotation has signal peptide region, we subtract the length of the signal peptide from the mutation site position, which results in an expanded annotation containing the adjusted sequence position. If the sequence position is not mentioned, we retain the original position of the mutation for the adjusted position. As shown in Fig. 7, the final gold standard annotation is recorded as < **APC_HUMAN**, *Ala, 1296, 1296, Val*; ***155255***, 10666372>. Quite often we observe that the mutation position mentioned in UniProtKB ID differed with the position mentioned in the text. This difference is mainly due to the length of the signal peptide region. In such cases, We adjust the mutation position by subtracting the length of the signal peptide region (39 in case of **GLCM_HUMAN**) from the one mentioned in the UniProtKB.

Since our focus was on association detection, we retained only those abstracts with at least one disease mention, one gene/protein mention and one mutation mention (tmVAR and MutationFinder). The data set was then split into two sets namely development (D) and test (T) sets. We used the development set to develop the MutD system and the test set for blind evaluation. We compared five variations of the system where the first two systems (S1 and S2) served as the baseline and the remaining three (S3, S4, and S5) utilized dependency graph traversal.

- S1 uses abstract-level co-occurrence.
- S2 uses sentence-level co-occurrence.
- S3 performs dependency graph traversal of a single sentence.
- S4 links dependency graphs across multiple sentences, using entity identifiers.
- S5 links dependency graphs, using entity identifiers, anaphoric terms, and trigger words.

### Evaluation metrics

We report the results in terms of precision, recall and F-measure where precision is a measure of system accuracy, recall its coverage, and F-measure, a harmonic mean of precision and recall. Let TP, FP, and FN be the number of correct, incorrect, and missed associations extracted by the system in comparison with the gold standard, respectively. The precision, recall, and F-measure are computed as follows:$\boldsymbol{Precision} = \frac{\boldsymbol{TP}}{\boldsymbol{TP}+\boldsymbol{F}\boldsymbol{P}},$ $= \frac{\boldsymbol{TP}}{\boldsymbol{TP}+\boldsymbol{F}\boldsymbol{N}},$ and $\boldsymbol{F}-\boldsymbol{Measure} = \frac{2\times \boldsymbol{Precision}\times \boldsymbol{Recall}}{\left(\boldsymbol{Precision}+\boldsymbol{Recall}\right)}$.

## Results and discussion

### Gold standard data set extracted from UniProtKB

There are 2,613 abstracts in total cited as literature evidence of curated protein mutation disease associations. Using state-of-the-art entity mention tools, we found 720 abstracts from 497 UniProtKB records with at least one disease mention, one mutation mention, and one gene/protein mention. We retrieved the title and abstract of the PubMed articles cited as evidence in the records using Batch Entrez (e-utils). The 720 abstracts were divided into development (576) and testing (144) with a ratio of 80:20. The development and test data sets contain 631 and 264 gold standard associations respectively.

Table 1 summarizes the ternary and binary relations contained in the development (D) and test (T) data sets. Note that not all binary relations are part of ternary relations. For example, there are 40 more protein-disease relations in the development data set but the underlying association is not about protein mutation disease information.

**Table 1:** Gold standard relation statistics (Development and test data set)

S. No	Relation	Data set	Total numbers
1	Protein-Mutation-Disease (PMD)	D	631
		T	264
2	Protein – Mutation (PM)	D	879
		T	388
3	Protein-Disease (PD)	D	671
		T	295

D – Development set; T – Test set

### System performance

In the task of relation extraction, we have two subtasks. 1) Named entity recognition and 2) Protein-Mutation-Disease relation extraction. We did not formally evaluate the named entity recognition and normalization in the current study due to our choice of considering UniProtKB as the gold standard. Evaluating named entity recognition and normalization against UniProtKB as Gold standard has the following limitations. PubMed (PMID) cited as a reference in UniProtKB against a particular protein often contain entities mentioned in an abstract. Besides we considered only those UniProtKB entries, which contain Protein-Mutation-Disease relation curated in UniProtKB. PubMed abstracts also contain other entity names, which may not be the focus of the current paper. Even though the entity mention detection is correct as per the text, since the PMID is not found in UniProtKB against those protein entries, such entities will be considered as inaccurate. Our estimation of Precision, Recall and F-measure will also not be accurate. For example, the abstract with PMID-21828135 is cited as a reference in 3 UniProtIDs (KDM6A_HUMAN, DNM3A_HUMAN, and EZH2_HUMAN). However, the abstract contains a sentence “TET2 mutations were present in 49 %, **ASXL1** in 43 %, **CBL** in 14 %, **IDH1/2** in 4 %, **KRAS** in 7 %, **NRAS** in 4 %, and **JAK2** V617F in 1 % of patients.” where additional protein names (in bold) were identified and normalized to respective UniProtIDs. While they are correct considering the information in the abstract, they will be wrong since they are not annotated in UniProtKB. Considering these factors, we did not perform evaluation for named entity performance and instead chose only the state of the art tools that are known to perform well in the detection and normalization of protein, mutation and disease entities in the text which have performed really well on shared tasks.

**Fig. 8:**
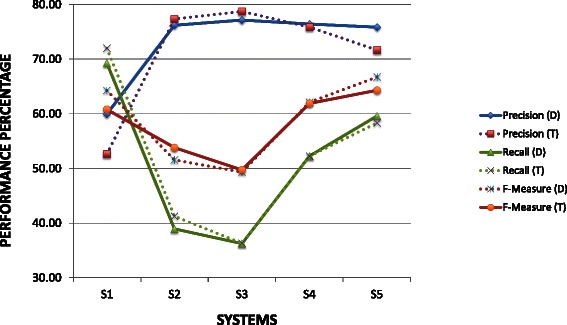
Performance trend of systems on development and test data set

**Fig. 9:**
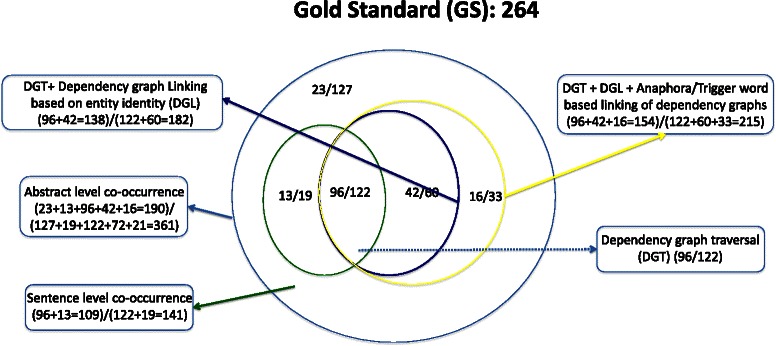
Comparison of performance of systems on test data

Table 2 lists the evaluation results on the development and test data sets of all the five system variants respectively. The columns under heading PMD in Table 2 refer to the performance of the ternary relation extraction among protein, mutation and disease. The performance metrics of binary relations such as protein-mutation (under heading PM), mutation-disease (under heading MD), and protein-disease (under heading PD) relations are also shown in Table 2. Figure 8 plots the performance of the ternary relation and Figure 9 intends to bring insights on the performance of individual systems and also highlight the gaps in their performance.

**Table 2:** Evaluation results on development (D) and test (T) data sets

Sys	Set	PMD			PM			MD			PD		
		P	R	F	P	R	F	P	R	F	P	R	F
S1	D	60.0	69.3	64.3	69.2	68.4	68.8	61.7	67.7	64.6	67.2	80.6	73.3
	T	52.6	72.0	60.8	65.5	71.4	68.3	57.0	70.9	63.2	61.1	80.9	69.6
S2	D	76.2	39.0	51.6	82.6	48.1	48.1	74.8	44.3	55.6	84.6	59.8	70.0
	T	77.3	41.3	53.8	76.3	43.0	55.0	67.8	43.6	53.1	74.7	61.4	67.4
S3	D	77.1	36.3	49.7	84.4	45.6	59.2	78.2	42.5	55.0	89.3	57.4	69.9
	T	78.7	36.4	49.7	79.1	38.9	52.2	77.2	41.8	54.3	76.7	59.7	67.2
S4	D	76.4	52.3	60.3	84.4	45.6	59.2	78.2	42.5	55.0	89.3	57.4	69.9
	T	75.8	52.3	61.9	79.1	38.9	52.2	77.2	41.8	54.3	76.7	59.7	67.2
S5	D	75.8	59.6	66.7	81.7	57.0	67.2	75.9	60.0	67.0	88.6	63.8	74.2
	T	71.6	58.3	64.3	76.8	58.0	67.2	74.8	59.3	66.1	76.2	67.7	71.7

**Sys** – Systems; **PMD –** Protein-Mutation-Disease relationships; PM – Protein-Mutation relationships; MD – Mutation-Disease relationships; PD – Protein-disease relationships; S1 (System1) – Abstract level co-occurrence; S2 (System2) – Sentence level co-occurrence; S3 (System3) – Sentence level dependency graph based traversal; S4 (System4) – Linking two dependency graphs based on entity identity; S5 (System5) – Linking two or more graphs based on anaphora resolution/trigger words.; P – Precision (in %); R – Recall (in %); F – F-measure (in %)

The abstract level co-occurrence (S1) has the highest recall, while the sentence level dependency graph traversal (S3) achieved the highest precision for extracting the ternary association between protein, mutation and disease. The maximum F-measure (achieved by S5) on the development and test sets is 66.7 % and 64.3 % respectively, close to 4 % percentage gain in the F-measure than abstract level co-occurrence (S1). Another interesting observation is that we found only 36 % of the ternary relations within a single sentence (S2).

We observed a trend similar to that of ternary relation extraction in the case of binary relation extraction. While the abstract level co-occurrence (S1) achieved the highest recall S3 achieved the highest precision, and S5 achieved the best F-measure for almost all the binary relation extraction tasks.

### Error analysis

**Fig. 10:**
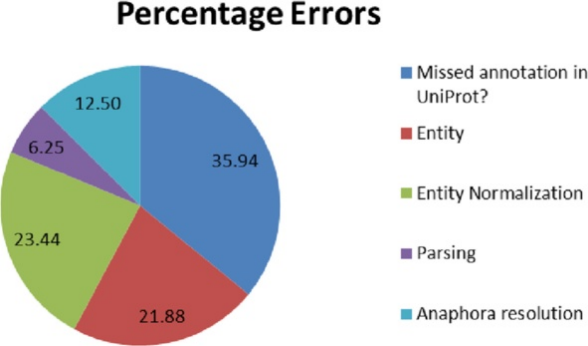
Percentage distribution of precision errors on test data set

**Fig. 11:**
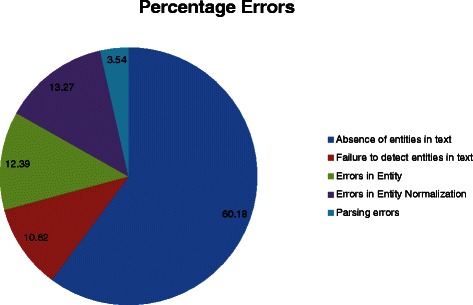
Percentage distribution of recall errors on test data set

In order to have better understanding of the system performance, we performed manual error analysis on the output of the best system (S5) using the test data set. Figures 10 and 11 illustrate the percentage distribution of both the precision and recall errors respectively.

There were 64 precision errors categorized in the following:

#### Absence of annotation in UniProtKB

We found nearly 23 (31 %) of the associations extracted by the system not captured by UniProtKB. For example, consider the following sentence from an example abstract (PMID - 9973276): “We conclude that the **APC** *I1307K* variant leads to increased adenoma formation and directly contributes to 3 %-4 % of all Ashkenazi Jewish ***colorectal cancer***.” The system extracted the ternary relation < **APC_HUMAN**, *Ile, 1307, Lys*, ***114500*** > from the above sentence. However, in UniProtKB for “APC_HUMAN” entry the annotation contains only protein-mutation binary relation (APC_HUMAN and Lys1307) leaving out the disease colorectal cancer.

#### Entity detection errors

Errors in entity detection are another significant source (~22 %) for precision errors. Such errors happen when there is an overlap in the text span between two entity classes. Consider the following sentence from an abstract (PMID - 11565064) “A homozygous *R279W* mutation was recently found in the *diastrophic dysplasia* sulfate transporter gene, **DTDST**, in a patient with ***MED*** who had a club foot and double-layered patella.” MutD correctly extracted the association among gene **DTDST**, mutation *R279W*, and disease ***MED***. However, the named entity recognition algorithm failed to recognize “**diastrophic dysplasia sulfate transporter gene**” as a single entity (gene/protein). Instead, MutD identified “***diastrophic dysplasia***” as a disease and normalized it to OMIM Id: **222600**. This led to the extraction of a false association between gene **DTDST**, mutation *R279W*, and disease ***diastrophic dysplasia***.

#### Entity normalization errors

Errors in entity normalization contribute significantly (23.44 %) to precision errors. For example consider the following sentence from an abstract (PMID: 18678517): “Thus, the current study identified the **DSG2**-*V55M* polymorphism as a novel risk variant for ***DCM*** associated with shortened desmosomes of the cardiac intercalated disc”. Three systems (S3, S4, and S5) gives the following output < **DSG2_HUMAN**, *Val, 55, Met*, ***115200|613424|613642***>. The gold standard annotation for this abstract reads as follows: <**DSG2_HUMAN**, *Val, 55, 56, Met*, ***612877***>. While all the fields matched correctly, none of the three OMIM Ids identified by the systems matched the one in the gold standard annotation.

#### Errors in linking dependency graphs through anaphora/trigger words

Linking dependency graphs through trigger words cause some of the errors. Consider the following two sentences from an abstract (PMID-21828135): “Mutational spectrum analysis of ***chronic myelomonocytic leukemia*** includes genes associated with epigenetic regulation: **UTX**, **EZH2**, and **DNMT3A**.” and “**TET2** mutations were present in 49 %, **ASXL1** in 43 %, **CBL** in 14 %, **IDH1/2** in 4 %, **KRAS** in 7 %, **NRAS** in 4 %, and **JAK2** *V617F* in 1 % of patients”. The trigger word “Mutational” (base form: Mutation) in the first sentence is linked to the point mutation *V617F* in the second sentence leading to incorrect associations among protein **JAK2**, mutation *V617F* and disease chronic myelomonocytic leukemia. Apart from these, dependency parsing also contributed to 3 % of the errors.

Totally, we identified 113 recall errors, detailed in the following:

#### Absence of entities in the text

Absence of entities in the text has been the major cause for the recall errors. Nearly 60 % of the errors are due to the absence of entity mentions in the abstract. For UniProtKB entry “**APC_HUMAN**” the variant “*CYS-1395*” is annotated to be involved in “***HEPATOBLASTOMA***” where the PMID “8764128” is cited as the literature evidence. However in the PubMed abstract there is no mention of any protein point mutation or variant. The algorithm correctly extracts the protein-disease relation. In UniProt entry **GLCM_HUMAN**, four variants (*SER-409*, *HIS-448*, *PRO-483* and *CYS-502*) are annotated to play a role in ***Gaucher’s disease*** with PMID “7627184” as the literature evidence. After subtracting the length of signal peptide region (39) from the residue positions, the modified variants are converted into *SER-370*, *HIS-409*, *PRO-444* and *CYS-463* respectively. While all the four modified variants and the disease are mentioned in the abstract, the protein is not even mentioned anywhere in the abstract, which results in 4 recall errors. Restricting our processing to only biomedical abstracts is a primary reason for this error.

#### Failure to detect entities

Failure to detect entities is another source for recall errors. Consider the following sentence from an abstract (PMID-1972019): “One mutation consists of a single-base substitution in three different codons: codon *444, Leu (CTG) to Pro (CCG)*; *codon 456, Ala (GCT) to Pro (CCT)*; and *codon 460, Val (GTG) to Val (GTC)*.” Both PubTator and Mutation-Finder (even with supplemented patterns) failed to recognize all the three mutation mentioned in the sentence resulting in recall errors.

#### Errors caused by precision errors

There are 41 precision errors also leading to recall errors. For example consider the following sentence: “Thus, the current study identified the **DSG2**-***V55M*** polymorphism as a novel risk variant for ***DCM*** associated with shortened desmosomes of the cardiac intercalated disc.” In this sentence, the disease “***DCM***” is not normalized to the OMIM ID annotated in the gold standard (UniprotKB). Such errors in entity normalization lead to both precision error and recall error.

The above detailed error analysis indicates 23 of the 64 precision errors are actual associations failed to be captured by database curators and 68 of the 113 recall errors are caused by the absence of associated disease entities in the abstract. If we neglect those errors, the adjusted precision, recall, and F-measure of S5 in association detection reach 82.3 %, 80.8 %, and 81.5 % respectively.

### Limitations and future directions

In this study, our predominant focus was to address the linguistic inference challenge from the text for database curation. We have not paid much attention to the initial steps (Step 1 and 2) and the expert inference challenge. Our approach to discourse level analysis is more effective when the scope of the problem under investigation is highly targeted like the one in this study. However, we believe that the issue of an expert inference cannot be completely addressed by text mining.

In addition, nearly 30-40 % of the gold standard ternary relations were missed by the system due to the absence of relevant entities in the abstract. One solution is to extend our system to process full text articles where we may find mention of more mutation and protein information in the text. As a logical next step, we plan to extend the system to process full text articles. However, removing the irrelevant associations will be one of the critical challenges that need to be addressed while we adapt our text mining approach to extract associations across sentences from full text articles. Also in this study, we predominantly focused only on protein point mutation disease associations. There are other significant variants at the gene and protein levels such as deletions and additions, which also play significant roles in diseases.

Our immediate next plan is to release both the web and RESTAPI versions of the MutD system. With the increased use of the next generation sequencing in clinical practice, linking clinical variants to literature mentions will help clinician interpret the impact of variants and guide them to take appropriate therapeutic intervention. We also plan to extend the system to extract drug-mutation-disease ternary relations from literature, which can be integrated with other knowledge sources such as ClinVar [2] may empower physicians to embrace individualized medicine.

## Conclusions

Our study attempts to measure the capability of a text mining system in automatically extracting database level annotations from biomedical texts. Our evaluation of the system against gold standard annotations extracted from curated database provides insight of the utility of text mining for database curation. From text mining perspective, this is the first attempt to effectively combine information from multiple sentences to extract ternary relations between protein, mutation and disease. Our approach to link dependency graphs across sentences using entity identity, anaphora and trigger words resulted in substantial performance improvement. Achieving a performance of 64.3 % of overall F-measure, which when further revised to 81.5 % after detailed error analysis demonstrates that our approach to some extent addressed the linguistic inference challenge faced by the use of text mining for database curation.

### Availability of supporting data

The protein-mutation-disease ternary association and binary association output for the MutD system for both the development and test data in BRAT annotation style is accessible at: http://ohnlp.org/index.php/MutD

## Abbreviations

OMIM: Online Mendelian Inheritance in Man
EMU: Extractor of Mutations
UIMA: Unstructured Information Management Architecture
UMLS: Unified medical language system
CTD: Comparative toxicogenomics database
MF: Mutationfinder
LVG: Lexical variant generator
NLM: National library of medicine
PMD: Protein mutation and disease
PD: Protein-disease
PM: Protein-mutation
MD: Mutation-disease
