# Erratum to: Text mining facilitates database curation - extraction of mutation-disease associations from Bio-medical literature

**DOI:** 10.1186/s12859-016-0974-0

**Published:** 2016-04-13

**Authors:** Komandur Elayavilli Ravikumar, Kavishwar B. Wagholikar, Dingcheng Li, Jean-Pierre Kocher, Hongfang Liu

**Affiliations:** Department of Health Sciences Research, Mayo Clinic College of Medicine, 200 First St SW, Harvick 3rd, Rochester, MN 55905 USA

Unfortunately, the original version of this article [1] contained an error. The family name of the last author (“Liu”) was captured as a given name, and “PhD” was captured as a family name. The correct first name is “Hongfang” and the correct family name is “Liu”. This is included correctly in the author list above.
